# A Case of Protracted and Difficult Labour, Followed by Sloughing of the Vagina, and Recto-Vaginal Septum

**Published:** 1844-02

**Authors:** John P. Anderson

**Affiliations:** Sparta, Alabama


Art. IV.—*A Case of protracted and difficult Labour, followed by Sloughing of the Vagina, and Recto-Vaginal Septum.* By Dr. John P. Anderson, of Sparta, Alabama.

The case about to be described cannot be regarded as unimportant, in a practical point of view. It admonishes, in a manner the most impressive, of the danger of suffering the head of the fœtus to remain long impacted in the pelvis, pressing with such force against the soft parts as to arrest the circulation in them, and finally destroy their vitality. The unhappy subject of the following case has consulted an eminent surgeon, in the hope that the injury might be repaired by an operation. She has not received much encouragement, and meantime the particulars of the case are laid before the profession. Should an operation be determined on, the result of it shall be communicated.

Mrs. B., æt. 24 years, was taken in labour in November, 1841; a physician was called in, who, on examination, ascertained that the waters had been discharged. On inquiry he was told that this had occurred some hours previously; otherwise all things promised well. Six hours passed without much increase of pain, but about the seventh hour it came on with such force that it was thought the child must be soon expelled, as the head had already descended far into the pelvis. In this situation things continued for thirty-eight hours, every effort at effecting delivery by natural means being made by the physician in vain. The powers of her system began now to give way, and as the medical attendant was reluctant to resort to embryotomy, although the child had been dead for some hours, the patient requested that I should be sent for, at a distance of forty-five miles. Many hours necessarily elapsed before I could reach her bed-side, and when I did so, I had the satisfaction to learn that her delivery had been accomplished two hours before. The head, of monstrous size, had been perforated, and removed, piece-meal, by her physician. Her life was thus preserved, but the most serious consequences resulted. The inflammation which followed, terminated in sloughing. A communication between the vagina and rectum was established, by the destruction of a large portion of the recto-vaginal septum, through which the fæces when fluid now find their way into the vagina, and are discharged involuntarily. She has perfect control over the urinary discharge, although the urethra was involved to the extent of five-eights of an inch in the sloughing. She menstruates regularly, and her general health is good. She is ready to submit to any surgical operation which affords any prospect of relief, preferring death to her present situation.

January, 1844.

